# Supplementary material for: Spontaneous and evoked neurotransmission are partially segregated at inhibitory synapses
Source: eLife. 2020 May 13;9:e52852. doi: 10.7554/eLife.52852 (PMC7250572; doi:10.7554/eLife.52852)
Supplement: Transparent reporting form [file elife-52852-transrepform.docx]

***eLife’s* transparent reporting form**

We encourage authors to provide detailed information *within their submission* to facilitate the interpretation and replication of experiments. Authors can upload supporting documentation to indicate the use of appropriate reporting guidelines for health-related research (see [EQUATOR Network](http://www.equator-network.org/%20)), life science research (see the [BioSharing Information Resource](https://biosharing.org/)), or the [ARRIVE guidelines](http://www.plosbiology.org/article/info:doi/10.1371/journal.pbio.1000412) for reporting work involving animal research. Where applicable, authors should refer to any relevant reporting standards documents in this form.

If you have any questions, please consult our Journal Policies and/or contact us: [editorial@elifesciences.org](mailto:editorial@elifesciences.org).

**Sample-size estimation**

- You should state whether an appropriate sample size was computed when the study was being designed
- You should state the statistical method of sample size computation and any required assumptions
- If no explicit power analysis was used, you should describe how you decided what sample (replicate) size (number) to use

Please outline where this information can be found within the submission (e.g., sections or figure legends), or explain why this information doesn’t apply to your submission:

The approach we used for this research is hypothesis free, we did not have prior knowledge of the type of result or values we were going to obtain or of the magnitude of the effect of blocking and unblocking GABA_A_Rs in a use-dependent manner. For that reason, prior statistical power analysis and sample-size estimation was not possible.

Nevertheless, the number of experiments and samples needed can be inferred from previous, similar experiments performed in our lab, using the same type of cultures and techniques (see Atasoy *et al*., 2008 and Sara *et al*., 2011). Assuming the reproducibility of experimental conditions and settings, we estimated that a minimum of 2 independent cultures with 3 coverslips per experimental group was enough for significance testing and finding the presence or absence of differences or tendencies among groups. Based on the variances and tendencies observed, in some cases the addition of extra experiments was decided.

**Replicates**

- You should report how often each experiment was performed
- You should include a definition of biological versus technical replication
- The data obtained should be provided and sufficient information should be provided to indicate the number of independent biological and/or technical replicates
- If you encountered any outliers, you should describe how these were handled
- Criteria for exclusion/inclusion of data should be clearly stated
- High-throughput sequence data should be uploaded before submission, with a private link for reviewers provided (these are available from both GEO and ArrayExpress)

Please outline where this information can be found within the submission (e.g., sections or figure legends), or explain why this information doesn’t apply to your submission:

The number of replicates (recordings obtained from independent coverslips) in each figure is represented in the corresponding legend. At least 2 independent cultures (using different rat litters, culture media and new batches of all experiment related buffers and drugs) were used for all experiments to correct for technical variances. Per culture, 3 to 6 coverslips were used per experimental group to include biological variances derived from differences in culture density and synapse density.

Outlier elimination was not performed during our studies.

**Statistical reporting**

- Statistical analysis methods should be described and justified
- Raw data should be presented in figures whenever informative to do so (typically when N per group is less than 10)
- For each experiment, you should identify the statistical tests used, exact values of N, definitions of center, methods of multiple test correction, and dispersion and precision measures (e.g., mean, median, SD, SEM, confidence intervals; and, for the major substantive results, a measure of effect size (e.g., Pearson's r, Cohen's d)
- Report exact p-values wherever possible alongside the summary statistics and 95% confidence intervals. These should be reported for all key questions and not only when the p-value is less than 0.05.

Please outline where this information can be found within the submission (e.g., sections or figure legends), or explain why this information doesn’t apply to your submission:

All statistical methods utilized are described at the end of the Methods section. For each experiment, N size, means, SEM and exact p values are reported in the corresponding figure legends.

(For large datasets, or papers with a very large number of statistical tests, you may upload a single table file with tests, Ns, etc., with reference to sections in the manuscript.)

**Group allocation**

- Indicate how samples were allocated into experimental groups (in the case of clinical studies, please specify allocation to treatment method); if randomization was used, please also state if restricted randomization was applied
- Indicate if masking was used during group allocation, data collection and/or data analysis

Please outline where this information can be found within the submission (e.g., sections or figure legends), or explain why this information doesn’t apply to your submission:

For each culture, all experimental groups were represented with the same number of replicates (for example, 4-5 coverslips per condition). Data were analyzed, and quality of recordings confirmed, by an experimenter blind to experimental condition.

**Additional data files (“source data”)**

- We encourage you to upload relevant additional data files, such as numerical data that are represented as a graph in a figure, or as a summary table
- Where provided, these should be in the most useful format, and they can be uploaded as “Source data” files linked to a main figure or table
- Include model definition files including the full list of parameters used
- Include code used for data analysis (e.g., R, MatLab)
- Avoid stating that data files are “available upon request”

Please indicate the figures or tables for which source data files have been provided:

Source data for each figure is provided as spread sheets, Figure 1 Source Data etc…
